# Effects of biotin on promoting anammox bacterial activity

**DOI:** 10.1038/s41598-021-81738-y

**Published:** 2021-01-21

**Authors:** Qinyu Li, Jinhui Chen, Guo-hua Liu, Xianglong Xu, Qian Zhang, Yijin Wang, Junli Yuan, Yinghao Li, Lu Qi, Hongchen Wang

**Affiliations:** Low Carbon Water Environmental Technology Research Center, School of Environment & Natural Resources, Renmin University of China, Beijing, 100872 China

**Keywords:** Biological techniques, Environmental sciences

## Abstract

Anaerobic ammonium oxidation (anammox) bacteria significantly improve the efficiency and reduce cost of nitrogen removal in wastewater treatment plants. However, their slow growth and vulnerable activity limit the application of anammox technology. In this paper, the enhancement of biotin on the nitrogen removal activity of anammox bacteria in short-term batch experiments was studied. We found that biotin played a significant role in promoting anammox activity within a biotin concentration range of 0.1–1.5 mg/L. At a biotin concentration of 1.0 mg/L, the total nitrogen removal rate (NRR) increased by 112%, extracellular polymeric substance (EPS) secretion and heme production significantly improved, and anammox bacterial biomass increased to maximum levels. Moreover, the predominant genus of anammox bacteria was Candidatus *Brocadia*.

## Introduction

Anaerobic ammonium oxidation (anammox) bacteria, a type of chemotrophic bacteria that uses bicarbonate as a carbon source, convert NH_4_^+^ and NO_2_^−^ into N_2_ under anaerobic or hypoxic conditions^1^. Owing to their low energy and organic carbon consumption, anammox improves the efficiency and reduces the cost of nitrogen removal in wastewater treatment^2,3^. Therefore, research in the field of novel biological processes is highly focused on anammox, and more than 100 full-scale anammox wastewater treatment plants (WWTPs) are in operation across the world^4,5^. Although anammox bacteria are widely distributed in natural ecosystems, their slow growth limits the application and promotion of anammox technology; anammox bacteria typically have a cell yield of 0.066 ± 0.010 mol C/mol NH_4_^+^, a cell-doubling time of 7–20 days, and a growth rate far lower than that of ordinary nitrifying bacteria^6,7^. In addition, anammox bacteria are sensitive to their external environment, including to factors such as illumination, temperature, pH, dissolved oxygen (DO), and substrate^8–11^.

Multifarious methods have been used to maintain anammox biomass or accelerate anammox activity to enrich anammox bacteria. Several solutions have been proposed, such as the use of different types of seed sludge and appropriate reactor configurations, all of which have advantages and limitations^12–21^. However, few studies have reported on the promotion of the anammox process by vitamin B, which is necessary for cell metabolism.

Biotin (vitamin B7) is a water-soluble vitamin that is essential for all organisms, from bacteria to humans^22^. In eukaryotic cells, biotin acts as a coenzyme, collectively known as carboxylase, which catalyzes key reactions in gluconeogenesis, amino acid catabolism, and fatty acid synthesis^23,24^. Biotin therefore promotes cell metabolism of sugar, fat, and protein and accelerates cell growth. Organisms also require biotin for carbon dioxide fixation, especially chemotrophic bacteria that use bicarbonate as a carbon source (e.g., anammox bacteria).

In this study, we assessed the short-term effects of different biotin doses on the biological activity and biomass of anammox bacteria. We also examined the effect of biotin on the anammox microbial community structure to determine the role of biotin in the anammox process. Our main aim was to identify the most feasible method for enhancing the growth and activity of anammox bacteria to reduce the start-up time of the anammox process in wastewater treatment.

## Materials and methods

### Synthetic wastewater

Synthetic wastewater containing substrates, bicarbonate, and trace elements was introduced into the experimental setup as the influent. The composition of the synthetic wastewater was similar to that described by Yang and Jin^25^. Ammonium and nitrite were supplied in the forms of NH_4_^+^–N and NO_2_^−^–N, respectively, and their initial concentrations in the influent were 80 mg/L and 104 mg/L, respectively. The influent pH was 7.5 ± 0.2 without the addition of acid or alkali. The DO of the reactor was below the limits of detection.

### Seed sludge

The anammox seed sludge with the diameter of 1.81 mm used in this study was obtained from a laboratory-scale anammox SBR reactor, with a sludge concentration of approximately 4500 mg/L and a nitrogen removal rate (NRR) of 41.2 mg N g^−1^ VSS d^−1^. The seed sludge was washed with phosphate buffer (140 mg/L KH_2_PO_4_ and 750 mg/L K_2_HPO_4_) to eliminate the influence of background nitrogen.

Each serum bottle was inoculated with 3 g of sludge (wet weight). The initial biomass concentration and the ratio of the volatile suspended solids (VSS) to the suspended solids (SS) of the anammox sludge were 0.785 ± 0.017 g VSS L^−1^ and 0.788 ± 0.003, respectively. The content of extracellular polymeric substances (EPS) was 145.2 ± 5.6 mg g^−1^ VSS, and the content of heme was 1.65 ± 0.3 mg g^−1^ VSS. The sludge was dominated by the anammox bacteria genus Candidatus *Jettenia asiatica*.

### Experimental procedures

**Figure 1:**
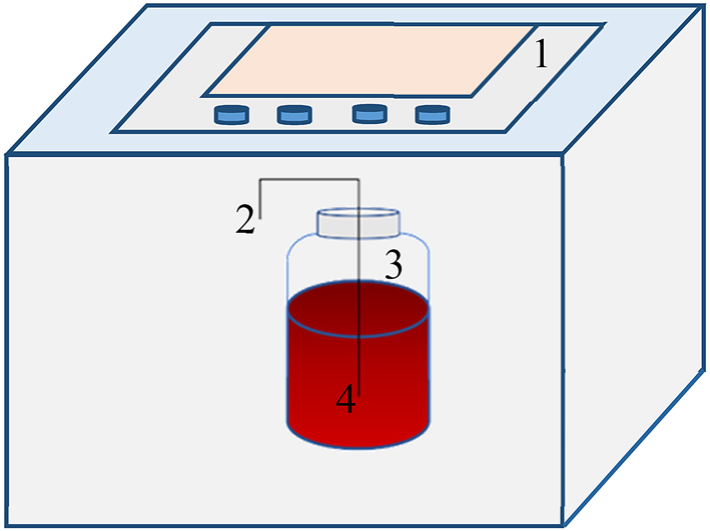
Schematic diagram of the reactor system: (1) Control panel for rotation speed and temperature, (2) sludge sample, (3) N_2_, and (4) serum bottle (with or without biotin).

Several 500 mL-serum bottles with 450 mL liquid-phase volume were used as the anammox reactors in this study, as shown in Fig. 1. To assess the performance of anammox with biotin, four serum bottles with different biotin concentrations were assigned as the four experimental groups. A serum bottle with the same conditions as the experimental groups but without biotin was used as the control. We wrapped the anammox reactors with aluminum foil to prevent light inhibition, and nitrogen was aerated into the reactor for 20 min to ensure the anaerobic state. The reactors were then placed in an air bath incubator (Changzhou, China) at 35 ± 1 °C, with a controlled rotation speed of 140 rpm.

### Analysis of anammox bacterial activity

Batch experiments were performed with the serum bottles to measure the NRR, EPS, electron transport system (ETS) activity, heme, and gene copy number of anammox bacteria. These measured parameters were then used to assess the short-term impact of biotin stimulation on anammox activity.

### Analytical methods

3 mL of supernatant as water samples were routinely collected every day with injector from the serum bottle, and filtered through 0.45 μm filter membranes to determine the NH_4_^+^–N, NO_2_^−^–N and NO_3_^−^–N concentrations using the standard methods described by APHA. The VSS of the granular sludge was also measured using standard methods^26^. EPS was extracted using the methods described by Yu et al.^27^ The main components—proteins (PN) and polysaccharides (PS)—were analyzed according to the methods described by Lowery et al.^28^ and Dubois et al.^29^ respectively. The ETS activities were measured by the method described by Trevors et al.^30^ The heme content was determined using the method described by Meng et al.^31^ Biotin concentrations were determined using the high-performance liquid chromatography (HPLC) method by Qi et al*.*^32^ Each of the experimental conditions was performed in triplicate to ensure experimental accuracy. The final data was the average of the triplicate results, and the standard deviation was calculated for subsequent analysis.

### DNA extraction and quantitative polymerase chain reaction (qPCR) assay

DNA extraction, and then quantitative PCR analysis were performed on the initial and the after six days of cultivation’s sludge under different concentrations of biotin. DNA was extracted from 0.5 g sample following the specifications of the FastDNA Spin Kit for Soil (MP, USA) for subsequent qPCR measurement. The DNA concentration was measured using a Nanodrop 2000 UV–Vis spectrophotometer (Nanodrop Technologies, Wilmington, DE, USA), and the gene copy number was calculated according to the concentration of plasmid DNA and the length of gene fragment. After obtaining the plasmid DNA, sterile water was used for continuous dilution to construct the standard curve. The amplification efficiency and correlation coefficient were greater than 105% and 0.98, respectively. The abundance of anammox bacteria was determined using an ABI 7000 real-time PCR instrument (Eppendorf, Germany) using the TransStart Green qPCR SuperMix (Transgen Biotech, Beijing, China). The forward and reverse primers were Amx 809f^33^ and Amx 1066r, respectively, and the primer information is shown in Table 1.

**Table 1:** Information of PCR primers used in this study.

Primer name	Sequence(5′-3′)
AMX809f	GCCGTAAACGATGGGCACT
AMX1066r	AACGTCTCACGACACGAGCTG

The total volume of the reaction system was 20 μL, including 10 μL TransStart Green qPCR SuperMix, 1 μL DNA template, 0.4 μL (10 mM) forward and reverse primers, and 8.2 μL ddH_2_O. The amplification procedure consisted of the following steps: 30 s at 94 °C, followed by 45 cycles of 10 s at 94 °C, 15 s at 55 °C, and 15 s at 72 °C.

## Results and discussion

### Nitrogen removal performance

**Figure 2:**
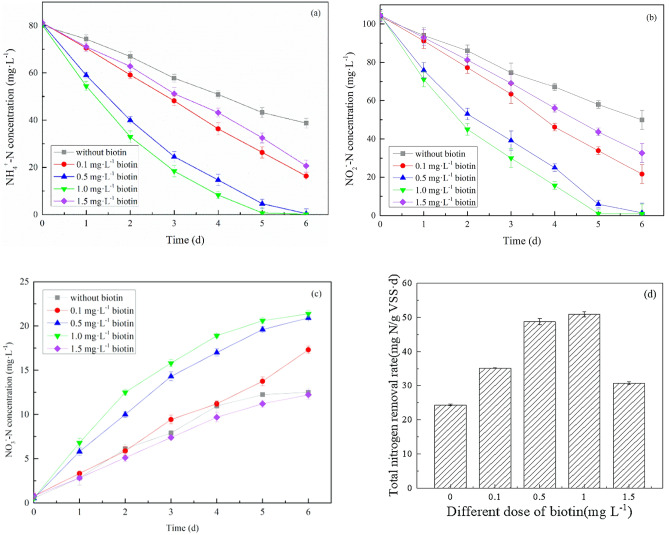
Concentrations of NH_4_^+^–N (**a**), NO_2_^−^–N (**b**), and NO_3_^−^–N (**c**) with time, and the total nitrogen removal rate at the end of the experiment (**d**).

The biotin concentrations added to each group was 0 mg/L (no biotin, the control group), 0.1 mg/L, 0.5 mg/L, 1.0 mg/L, and 1.5 mg/L, and the results of the experiments are shown in Fig. 2a–d.

Compared with the control group, we observed a significant increase in the total nitrogen removal efficiency after the addition of biotin (Fig. 2a–c). After six days of cultivation, NH_4_^+^–N and NO_2_^−^–N concentrations in the control group declined from 80.0 to 38.8 mg/L and from 104 to 49.6 mg/L, respectively, and the NO_3_^−^–N concentration increased to 12.5 mg/L. The △NH_4_^+^–N: △NO_2_^−^–N: △NO_3_^−^–N ratio was 1: 1.31: 0.30, which was approximately in accordance with the anammox physicochemical reaction formula, 1 NH_4_^+^  + 1.32 NO_2_^-^ + 0.066 HCO_3_^-^ + 0.13 H^+^ → 1.02 N_2_ + 0:26 NO_3_^−^ + 0.066 CH_2_O_0.5_N_0.15_ + 2.03 H_2_O. In the batch tests, the NH_4_^+^–N and NO_2_^−^–N concentrations declined to 1.0 and 0.3 mg/L, respectively, at a biotin dosage of 0.5 mg/L, and to 0.5 mg/L and 0.1 mg/L at a biotin dosage of 1.0 mg/L. As the concentration of total inorganic nitrogen changed from 185.6 mg/L to 21.9 mg/L, the minimum concentrations of NH_4_^+^–N and NO_2_^−^–N and the maximum concentration of produced NO_3_^−^–N (21.3 mg/L) were observed in the 1.0 mg/L biotin group; this suggests maximum bacterial activity and optimal biotin concentrations at 1.0 mg/L biotin. The concentrations of biotin during the cultivation process are shown in Table 2. The results indicated different amounts of biotin loss during the experimental process. This may be attributed to the cell utilization of biotin. Both biotin and enzymes contribute to the metabolic processes of the three main nutrients in cells. For example, the enzyme pyruvate carboxylase uses biotin as a coenzyme in the reaction between pyruvate and succinate during gluconeogenesis. In the process of fatty acid synthesis, biotin acts as a coenzyme of acetyl-CoA carboxylase to catalyze acetyl-CoA and produce malonate-CoA^34^. Biotin plays an important role in protein and purine synthesis as well as the catabolism of tryptophan and leucine. It is also a dominant growth factor for certain microorganisms^35^.

**Table 2:** Biotin concentrations during the cultivation process in the experimental system.

Time(day)	Biotin concentration (mg/L)				
	0.0	0.1	0.5	1.0	1.5
1	0.0	0.0984	0.500	1.00	1.50
6	0.0	0.00450	0.0197	0.0179	0.00990

The removal rate of NH_4_^+^–N and NO_2_^−^–N gradually reduced during the experiment at initial biotin concentrations of 0.5 mg/L and 1.0 mg/L (Fig. 2a,b). This can also be attributed to the gradual decrease in biotin concentration, which reduced its anammox bacterial activity stimulation.

Under the optimum biotin dose (1.0 mg/L), the average NRR was 50.9 mg N g^−1^ VSS day^−1^, and the NRR was 112% higher than the control group (Fig. 2d). We also observed no prominent enhancement of anammox bacterial activity at a biotin dose of 1.5 mg/L. Combined with the analysis of bacterial community structure, we infer the consumption of biotin by heterotrophic bacteria (*Thaurea* genus of *Proteobacteria* phylum, *Micromonospora* genus of *Actinobacteria* phylum, etc.) in the reaction system, which negatively impacted biotin stimulation on anammox bacterial activity. This suggests that higher biotin concentrations may have promoted the growth of heterotrophic bacteria and influenced the original anammox system. These results highlight the need for optimal concentrations of biotin within 0.1 to 1.5 mg/L to enhance anammox bacterial activity appropriately. The NRR in this study was higher than that of other previous studies on nitrogen removal performance by anammox bacteria^36–39^, which suggests that biotin addition can effectively enhance anammox bacterial activity.

### EPS analysis

**Figure 3:**
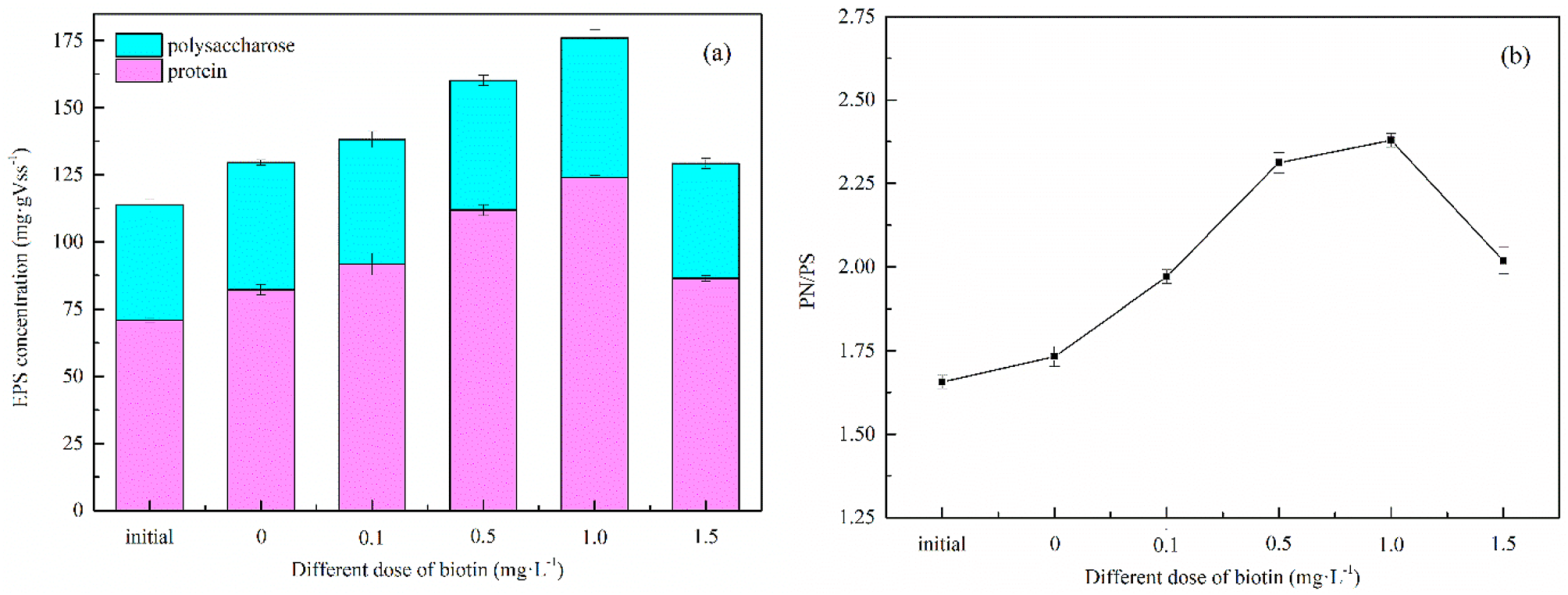
Changes of EPS concentrations (**a**) and PN/PS ratio (**b**).

Anammox bacteria tend to secrete a significant amount of EPS during their growth process; this largely impacts the physical and chemical properties of microbial polymers, such as flocculation, stability, and adsorption, and thus influences the activity of anammox bacteria sludge^40,41^. EPS is a metabolic product that accumulates on the surface of bacterial cells; the layer of PN and PS, the main components of EPS, protects cells from the external environment^42^. In this study, we assessed the effect of biotin on anammox bacterial activity by detecting changes in EPS concentrations (Fig. 3).

The initial concentrations of PN and PS in the sludge were 70.9 and 42.8 mg gVSS^−1^, respectively. Without biotin, the total EPS of the control group increased by 14.1% after 6 days of culture, and its content reached 130 mg gVSS^−1^. Compared with the control group, EPS significantly increased by 21.5%, 40.8%, and 54.8% at biotin concentrations of 0.1, 0.5, and 1.0 mg/L, respectively. PN, PS, and EPS contents were the highest (124, 52.1, and 177 mg gVSS^−1^, respectively) at a biotin concentration of 1.0 mg/L. However, the total EPS was 132 mg gVSS^−1^ (86.4 mg gVSS^−1^ PN, 42.8 mg gVSS^−1^ PS) at a biotin dose of 1.5 mg/L, which was similar to the results of the control group (without biotin). We therefore infer the consumption of biotin by heterotrophic bacteria in the reaction system under this biotin dosage. Moreover, heterotrophic bacteria may also use the original EPS in the system as a carbon source during their own proliferation and growth.

EPS can promote the physical bridging effect in microbial cell aggregation by changing the negative charge on the surface of bacteria; it can also help to form the porous structure of sludge^43^ (sludge granulation), which improves the stability of the system and the activity of anammox bacteria^44^. We found that higher biotin dosages (from 0.1 to 1.0 mg/L) increased the production of EPS by anammox bacteria. Moreover, a biotin dosage of 1.0 mg/L had the strongest enhancing effect on EPS production. Gao et al. showed that highly active anammox bacteria secreted more EPS^45^, which is consistent with the positive correlation between EPS yield and anammox bacterial activity in this experiment. The PN/PS ratio is also an important indicator of sludge properties, which reflects the sedimentation property of microorganisms^46^. In our study, the PN/PS ratio of each sample was higher than that of the control group. PN/PS gradually increased and reached a maximum value of 2.38 at 1.0 mg/L biotin; however, the sludge performance showed no significant changes. The change in PN/PS may have therefore been insufficient to exert a significant impact on sludge performance.

### ETS activity analysis

**Figure 4:**
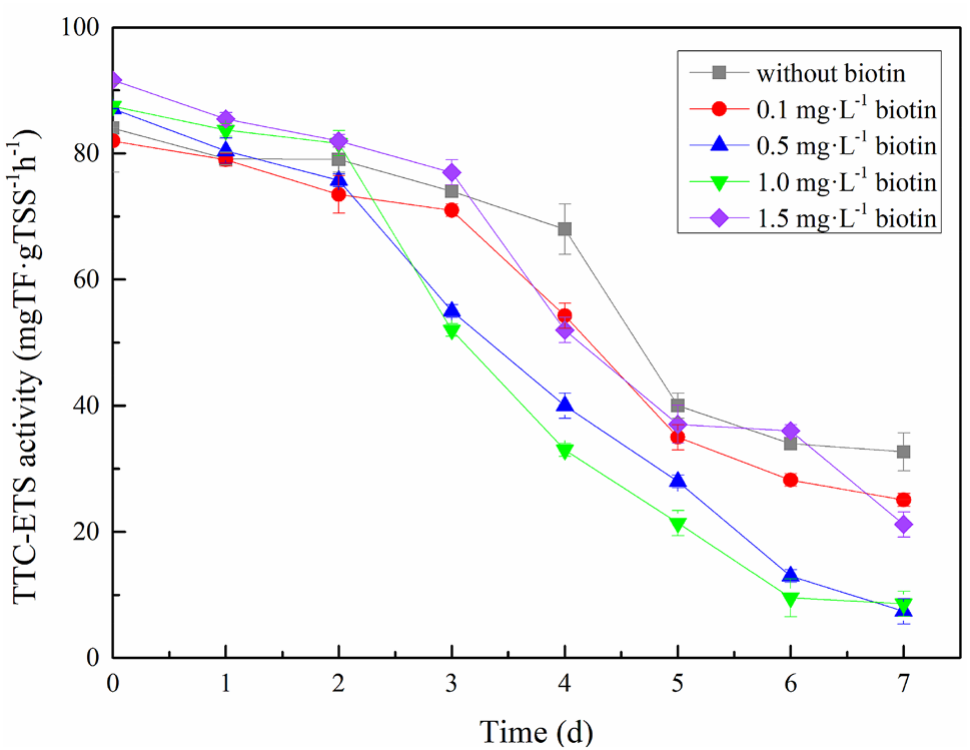
TTC-ETS activity under various biotin doses.

During aerobic or anaerobic microbial respiration, electrons are passed through a series of electron carriers to the final electron acceptor to remove organic pollutants. These electronic carriers are arranged in the order of increasing electron affinity, which constitutes the ETS. The ETS activity of sludge is an index of the microbial ability to reduce the number of artificial electron acceptors (redox potential is lower than that of natural ultimate electron acceptors) under certain conditions^47^. Measurements of electron transfer rates on the respiratory chain of sludge microorganisms can therefore indirectly characterize the microbial activity in sludge^30^. More hydrogen (electrons) transferred to the final electron acceptor through the peroxidation respiration chain per unit time infers a faster microbial respiration rate and higher biological activity in sludge, and vice versa. Triphenyltetrazolium chloride (TTC) is often used as an artificial electron receptor to detect ETS activity^48^. TTC-ETS activity is therefore the ETS activity detected by TTC and is shown in Fig. 4.

We observed a decrease in TTC-ETS activity with decreasing substrate before the complete consumption of the internal substrate. Following complete substrate consumption after 6 days with 0.5 and 1.0 mg/L biotin addition, TTC-ETS activity rapidly decreased to its lowest level and remained stable, indicating the complete removal of NH_4_^+^–N and NO_2_^−^–N in the system. The decrease in TTC-ETS activity in the 0.5 and 1.0 mg/L biotin groups occurred earlier and was more significant than the other biotin groups; this suggests a more rapid substrate consumption and higher biological activity in the anammox sludge of these two groups. Our TTC-ETS findings therefore indicate the removal of substrate (NH_4_^+^–N, NO_2_^−^–N), which is in line with our results on nitrogen removal performance. This observation is consistent with previous studies^49,50^ that reported a positive relationship between substrate (NH_4_^+^-N) removal and TTC-ETS activity in sludge. However, TTC-ETS activity in the 0, 0.1, and 1.5 mg/L biotin groups did not reach the minimum value, indicating incomplete substrate reaction and consumption. Moreover, the electron transfer process was still active, inferring low biological activity in the anammox sludge of these three groups.

### Heme analysis

Heme concentration was positively correlated with the nitrogen removal performance of anammox sludge, which directly reflects the sludge anammox activity^51^. In this study, we used a fluorescence spectrometer to measure the fluorescence intensity at an excitation wavelength of 405 nm and an emission wavelength of 500–700 nm. The characteristic peak fluorescence intensity of heme ferrous was detected at 601.4 nm and 655 nm. We then calculated the heme ferrous content in the anammox sludge from the standard curve.

As shown in Table 3, heme content also increased with increasing biotin concentration within 0–1.0 mg/L biotin. The 1.0 mg/L biotin group showed the highest heme content at 2.67 mg gVSS^−1^, which increased by 43.8% of the initial value at 1.86 mg gVSS^−1^, indicating the highest biological activity. The heme content in the 1.5 mg/L biotin group (2.39 mg gVSS^−1^) was lower than that of the 1.0 mg/L biotin group, which is consistent with the results of nitrogen removal performance. Previous studies^51,52^ reported a positive correlation between heme concentration and NRR at 35 °C. Moreover, Chen et al. observed an increase in heme concentration with increasing nitrogen removal capacity during the start-up period of an anammox process^53^. These results are in agreement with the observed positive correlation between heme and NRR in this study. NRR reached the highest value of 50.9 mg N g^−1^ VSS day^−1^ in the 1.0 mg/L biotin group, which suggests that 1.0 mg/L biotin is the optimum concentration (within the selected gradient) for the growth and activity of anammox bacteria.

**Table 3:** Fluorescence intensity and concentration of heme.

Sample	601.4 nm (A.U.)	655 nm (A.U.)	601.4 nm (mg/gVSS)	655 nm (mg/gVSS)	average (mg/gVSS)
initial	190	129	1.82	1.90	1.86
0 mg/L	207	137	2.24	2.19	2.21
0.1 mg/L	215	146	2.45	2.46	2.46
0.5 mg/L	217	147	2.49	2.49	2.48
1.0 mg/L	231	147	2.83	2.52	2.67
1.5 mg/L	213	143	2.40	2.38	2.39

### qPCR results

**Figure 5:**
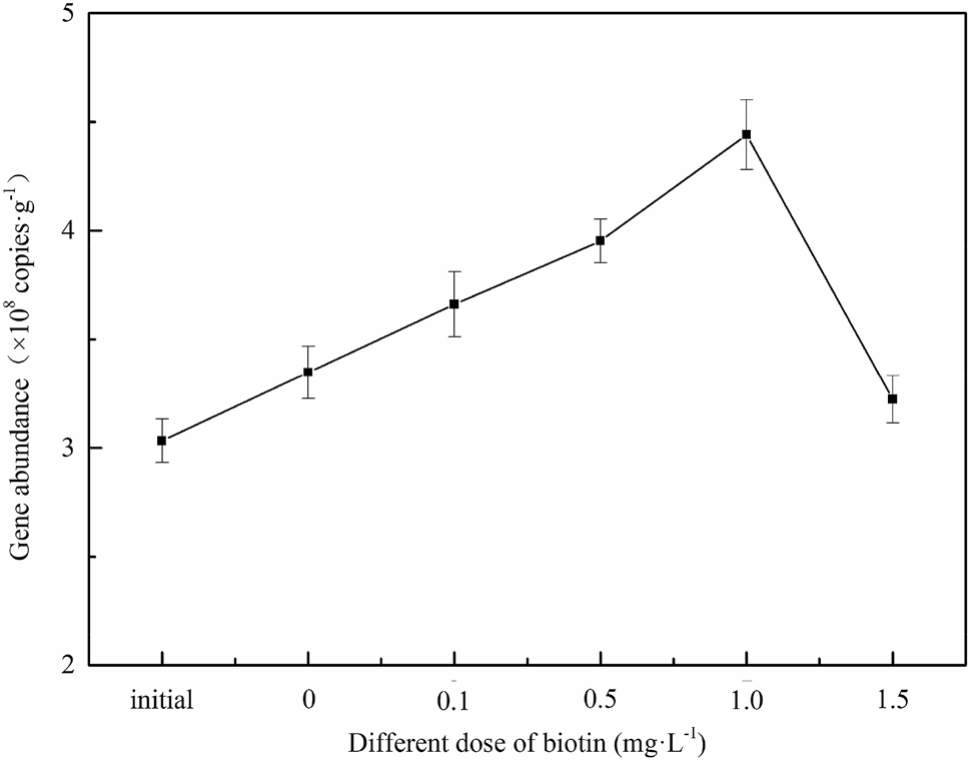
Anammox bacteria gene abundance under different biotin doses.

We conducted real-time qPCR to determine the gene abundance of anammox bacteria before and after biotin addition, as shown in Fig. 5. The abundance of anammox bacteria gradually increased with increasing biotin concentration. Moreover, the copy number of the functional genes of anammox bacteria in the 1.0 mg/L biotin group showed the most significant increase from 3.03 × 10^8^ to 4.44 × 10^8^ copies/g dry sludge (or a 46.5% increase). Biotin addition therefore notably accelerated the growth of anammox bacteria. This may be owing to biotin’s nitric oxide (NO)-like function, which increases the intracellular concentration of the second messenger cGMP (through the activation of the soluble form of the enzyme guanylate cyclase (sGC)) and improves the rate of cell division^54,55^. However, the abundance of functional genes of anammox bacteria in the 1.5 mg/L biotin group (3.22 × 10^8^ copies/g dry sludge) was similar to that of the non-biotin group (3.35 × 10^8^ copies/g dry sludge); this was likely linked to the consumption of biotin by heterotrophic bacteria in the reaction system, which inhibited the biotin stimulation of anammox bacteria.

### Community structure analysis

To investigate the influence of biotin on the anammox bacteria community structure, the anammox sludge in the reactor was sampled for high-throughput sequencing. Samples S1–S5 represent the anammox sludge with biotin additive dosages of 0, 0.1, 0.5, 1.0, and 1.5 mg/L, respectively; S6 was the original sludge without biotin addition.

The microbial community has been in a dynamic process for a long period, and the microbial community structure may have changed with the addition of external substances. To date, five genera of anammox bacteria have been reported—*Kuenenia, Brocadia, Jettenia, Scalindua, and Anammoxoglobus*—all of which are affiliated with *Planctomycetes*^56^. According to the diversity index of microorganisms in Table 4, the microbial diversity in the five groups of reactors with biotin was significantly lower than that of the original sludge. S6 showed the highest microbial diversity index, with various bacteria occupying a stable ecological niche in the original system, indicating ecosystem stability. The microbial diversity index of the biotin-stimulated samples showed a decreasing trend, with S4 (with 1.0 mg/L biotin) showing the lowest diversity.

**Table 4:** Sludge sample sequencing information and microbial diversity index.

Sample Name	Observed species	Shannon	Simpson	Chao1	ACE	Goods coverage
S1	460	6.04	0.963	478	485	0.999
S2	420	4.57	0.808	470	452	0.998
S3	460	5.42	0.891	494	493	0.998
S4	433	5.42	0.922	453	462	0.999
S5	467	6.09	0.961	519	501	0.998
S6	537	6.68	0.976	557	566	0.999

**Figure 6:**
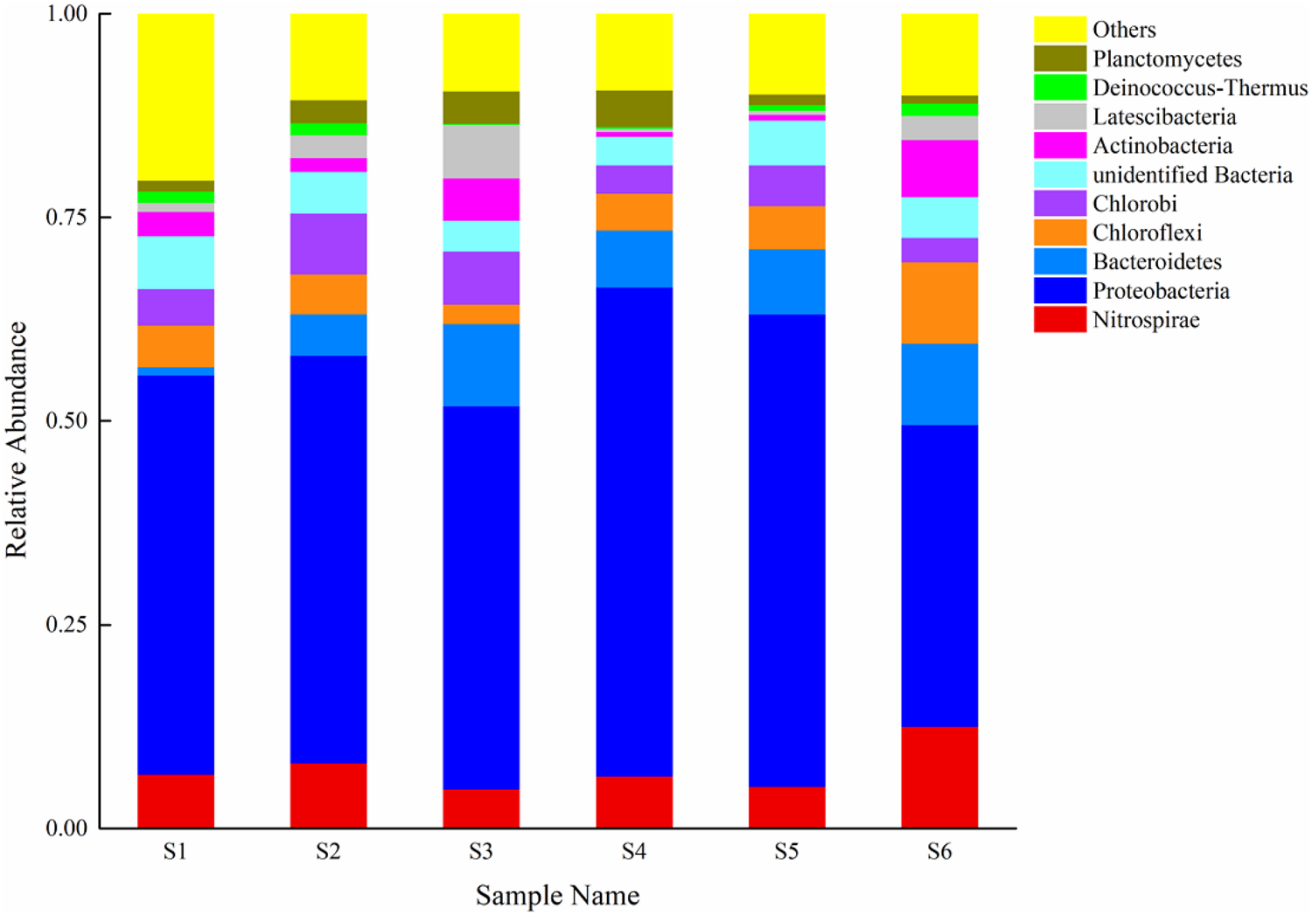
Taxonomic classification (phylum level) of the bacterial communities in each sludge sample (S1–S5).

A histogram of the 10 most abundant species in each sample (based on 16S rDNA amplicon sequencing at the phylum classification level) is shown in Fig. 6.

The bacterial community structure of the sludge in the reactor changed significantly following the addition of different biotin doses (Fig. 6). In the original sludge, the main bacteria were *Nitrospirae*, *Proteobacteria*, *Bacteroidetes*, and *Chloroflexi*. *Proteobacteria* accounted for the largest proportion in all six samples, ranging from 61.7 to 77.9%. The number of *Planctomycetes* bacteria increased gradually with increasing biotin, indicating the positive effect of biotin on *Planctomycetes* growth and proliferation. Nitrogen-removing microorganisms were mainly from *Proteobacteria*, *Nitrospiare,* and *Planctomycetes*, which accounted for 50.5%–70.9% of the total bacteria after biotin addition. Nitrogen removal efficiency therefore improved with biotin addition, reaching 88.4% at 1.0 mg/L biotin.

Three major genera were detected in *Planctomycetes*, of which Candidatus *Brocadia* and Candidatus *Jettenia* were anammox bacteria. The proportions of Candidatus *Brocadia* and Candidatus *Jettenia* in the inoculated sludge were 1.3% and 1.7%, respectively. After cultivation with biotin, Candidatus *Brocadia* gradually increased to 1.9% (exceeding Candidatus *Jettenia* at 0.1%) and was therefore the dominant genus of anammox bacteria in the reactor. Candidatus *Jettenia* can only live at low nitrogen loading rates, whereas Candidatus *Brocadia* has a higher proliferation rate at high nitrogen loading rates^57^. The change in nitrogen loading rate may therefore be the cause for the change in the dominant anammox strains. However, further analysis is required to elucidate the cause of bacterial species changes during the anammox enrichment process.

## Conclusions

We found that the addition of biotin within a limited gradient promoted biological activity and enhanced the biomass of anammox bacteria in short-term batch tests. The anammox activity showed the most significant improvement in the 1.0 mg/L biotin group, with a NRR increase of 112% compared with the control. This group also produced the highest concentration of total EPS and heme, with an increase of 54.8% and 43.8%, respectively, compared with their initial contents. The largest decrease in TTC-ETS activity was also observed in the 1.0 mg/L biotin group, indicating the highest biological activity in the anammox sludge of this group. The maximum abundance of anammox bacteria also increased by 46.4%. The predominant anammox bacteria were Candidatus *Brocadia*, whereas the abundance of Candidatus *Jettenia* gradually decreased with increasing biotin supplementation.
